# Automatic landmark detection and mapping for 2D/3D registration with BoneNet

**DOI:** 10.3389/fvets.2022.923449

**Published:** 2022-08-18

**Authors:** Van Nguyen, Luis F. Alves Pereira, Zhihua Liang, Falk Mielke, Jeroen Van Houtte, Jan Sijbers, Jan De Beenhouwer

**Affiliations:** ^1^Imec—Vision Lab, Department of Physics, University of Antwerp, Antwerp, Belgium; ^2^Departamento de Ciência da Computação, Universidade Federal do Agreste de Pernambuco, Garanhuns, Brazil; ^3^Department of Biology, University of Antwerp, Antwerp, Belgium

**Keywords:** 2D/3D registration, landmark-based registration, pose estimation, automatic landmark detection, deep learning

## Abstract

The 3D musculoskeletal motion of animals is of interest for various biological studies and can be derived from X-ray fluoroscopy acquisitions by means of image matching or manual landmark annotation and mapping. While the image matching method requires a robust similarity measure (intensity-based) or an expensive computation (tomographic reconstruction-based), the manual annotation method depends on the experience of operators. In this paper, we tackle these challenges by a strategic approach that consists of two building blocks: an automated 3D landmark extraction technique and a deep neural network for 2D landmarks detection. For 3D landmark extraction, we propose a technique based on the shortest voxel coordinate variance to extract the 3D landmarks from the 3D tomographic reconstruction of an object. For 2D landmark detection, we propose a customized ResNet18-based neural network, BoneNet, to automatically detect geometrical landmarks on X-ray fluoroscopy images. With a deeper network architecture in comparison to the original ResNet18 model, BoneNet can extract and propagate feature vectors for accurate 2D landmark inference. The 3D poses of the animal are then reconstructed by aligning the extracted 2D landmarks from X-ray radiographs and the corresponding 3D landmarks in a 3D object reference model. Our proposed method is validated on X-ray images, simulated from a real piglet hindlimb 3D computed tomography scan and does not require manual annotation of landmark positions. The simulation results show that BoneNet is able to accurately detect the 2D landmarks in simulated, noisy 2D X-ray images, resulting in promising rigid and articulated parameter estimations.

## 1. Introduction

Understanding an animal's 3D kinematics has long been a topic of interest in veterinary research (1–3). Such motions can be reconstructed by aligning a 3D reference model to a series of X-ray projection images, which is generally known as 2D/3D registration (4). Intensity-based and feature-based methods are the two major approaches of 2D/3D registration (5, 6).

Intensity-based 2D/3D registration methods rely on the pixel/voxel gray values to reconstruct 3D poses of an object from 2D images with reference to a 3D model. A similarity measure (SM) is computed as intensity or gradient difference between the acquired 2D projections of the object and simulated projections of the 3D reference model (7–10). The object's pose parameters are then estimated by minimizing the SMs. These methods, however, usually require a good initialization of the pose parameters to avoid the optimizations converging to local minima. Khamene et al. (9) dealt with this problem by pre-calibrating the system geometry, and Varnavas et al. (10) pre-registered the target pose to a broad range of possible poses within a 2D library generated from a 3D computed tomography (CT) object model. The intensity-based registration accuracy also depends on the SM robustness, which is sensitive to the different gray value distributions across image modalities or acquisition setups. To tackle this issue, Birkfellner et al. (11) presented stochastic rank correlation as an intensity invariant SM with stochastic sampling while Munbodh et al. (12) calculated SM from Poisson and Gaussian distribution models of CT and X-ray images, respectively. Intensity-based methods also involve computationally expensive simulations of the 2D radiographs during parameter estimation. Finally, projecting a 3D CT volume onto a 2D plane suffers from the loss of depth information (13).

Feature-based registration techniques circumvent the computational cost of the intensity/gradient-based methods (6, 14). The object's geometric features, such as curves, surfaces, landmarks, etc., are extracted and mapped to the corresponding features on the 3D model to obtain the orientation and translation parameters of the object. Feature-based registration methods allow fast estimation of the pose parameters as no reconstruction or simulation of the 2D radiographs is required during optimization. Baka et al. (14) and Ito et al. (15), for instance, estimated the 3D motion model of an object by matching the simulated and measured object curves. However, obtaining corresponding curves proved to be challenging as they are subject to the image's dynamic range and contrast. Geometrical landmarks have been suggested to represent a bone for kinematics registration (16–18). Joint kinematics are usually modeled as a combination of articulated transformations of individual bones, and geometric landmarks are manually annotated by experienced operators. Hasse et al. (16) applied an active appearance model to track the anatomical landmarks of birds of different species. However, manual landmark annotation and tracking relies on the acquisition setup, and expert experience, such as that from a radiologist. Annotating the landmarks or automatically detecting them while maintaining the mapping for registration is non-trivial, raising the need for an automated and robust landmark detection method. Cai et al. (19) automated the landmark candidate selection based on Harris corner detection, which relies on local intensity of image patches and does not account for global correlations, reducing its robustness.

Recently, following the advance of deep learning techniques in solving a wide range of computer vision problems, deep networks have been proposed for automated landmark detection (20–23). Since deep learning models can learn and generalize abstract features from a large amount of data, they are robust for landmark detection. Liao et al. (20) applied a Siamese network to detect a set of points of interest (POIs) in an input X-ray image. Although the POIs selected from CT models by a random method result in convergence during training, the randomization might induce overlapping POIs in 2D projections. DeepLabCut (21) is a well-known deep network for automatic landmark detection and tracking in optical images, which requires relatively few (hundreds) of labeled images to fine-tune a ResNet-based neural network for a new type of data or object. The method was applied to marker tracking on an X-ray videography scene that followed the positions of the markers attached to animals during their feedings (22). However, DeepLabCut requires manual landmark annotation in video frames that are used to generate the training dataset. This procedure is non-trivial and prone to human errors, especially for biological X-ray data with multiple landmarks usually distributed densely on a single bone. PVNet (23) is another deep learning model recently proposed to automatically detect nine 2D landmarks in optical images. To tackle the complexity of 3D pose reconstruction from a single X-ray radiograph of a biological object, PVNet requires customizations for inference of more landmarks and application to X-ray images.

In this paper, we introduce a comprehensive, automatic landmark detection and tracking method using a deep neural network named BoneNet, for 2D/3D registration of X-ray fluoroscopy images with a 3D CT reference model. It relies on a simulation module to generate well-labeled training, validation, and test datasets to eliminate human errors in manual landmark annotation. The module simulates different articulated poses of an animal using a single high-resolution 3D CT model. 3D reference landmarks are then extracted automatically using the same CT model. To this end, we present two techniques based on a shortest coordinate variance to define two types of 3D landmarks: bounding and SIFT (Scale-Invariant Feature Transform) landmarks. The bounding landmarks (23) are selected from the object voxels, while the SIFT landmarks are obtained from 3D SIFT keypoints extracted for conventional image matching (24). Finally, a deep neural network, inspired by PVNet (23), is trained to detect 2D landmarks in fluoroscopy images automatically. The network architecture is customized to better extract abstract features from complex X-ray image data with more landmarks.

The paper is structured as follows. Section 2 presents our proposed methodology for 3D landmark extraction from the reference model of the object, along with the process to detect the 2D landmarks accurately with deep learning and to reconstruct the object poses using a least-squares optimizer (25, 26). A technique to simulate realistic 3D articulated motions of the object is also presented in this section. Then, experiments using simulation data to validate the feasibility of our proposed method are discussed in Section 3. Finally, further discussion and the conclusion are presented in Sections 4 and 5, respectively.

## 2. Methods

### 2.1. Locomotion and geometry parameterization

**Figure 1:**
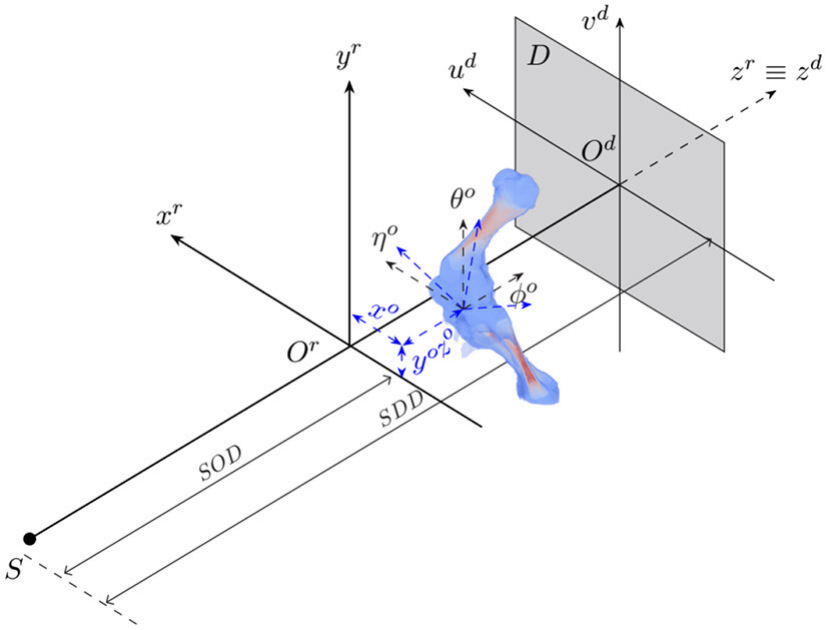
The geometry of a cone-beam acquisition system with an X-ray source *S* and a detector plane *D*. Object position and orientation with reference to the acquisition coordinate system *O*^*r*^*x*^*r*^*y*^*r*^*z*^*r*^ are represented by six parameters {*x*^*o*^, *y*^*o*^, *z*^*o*^, θ^*o*^, ϕ^*o*^, η^*o*^}.

The animal motion during an X-ray scan can be described by a rigid transformation for representing its position and orientation with respect to the acquisition geometry, and articulated transformations of bones of interest and soft tissues relative to individual joints. 2D/3D registration involves both estimation of the animal's rigid transformation in the acquisition geometry and its 3D pose with respect to the reference model. Figure 1 shows the geometry of an X-ray cone-beam acquisition system that is used to acquire animal fluoroscopy images. The system is assumed to be calibrated in advance. In other words, the perpendicular projection of the X-ray source on the detector plane *O*^*d*^*u*^*d*^*v*^*d*^ coincides with the detector center *O*^*d*^. Also, the distances from the source to the acquisition system's isocenter (*SOD*) and the detector plane (*SDD*) are assumed to be known. In this setting, the 3D position and orientation of the animal are represented by six parameters {*x*^*o*^, *y*^*o*^, *z*^*o*^, θ^*o*^, ϕ^*o*^, η^*o*^} about three axes (*x*^*r*^, *y*^*r*^, *z*^*r*^).

**Figure 2:**
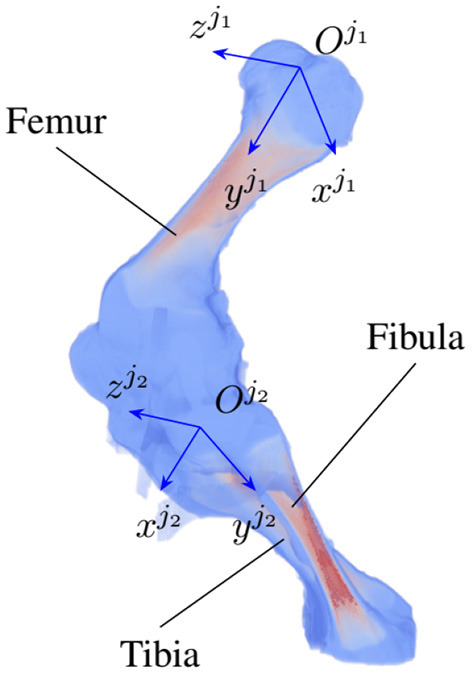
An example of joint coordinate systems of a piglet hindlimb with two major bones (femur and tibia). Each local coordinate system is represented by three axes $\left\{x^{j_{i}},y^{j_{i}},z^{j_{i}}\right\}$ which are the vertical, longitudinal, and transverse axis of joint *j*_*i*_, *i* = 1, 2, respectively.

As the locomotion of an animal involves a chain of contraction and relaxation of different muscles and tendons (27), the articulated transformation of bone *j*_*i*_ can be modeled by rotations around the bone's principal axes. The axes include the vertical $x^{j_{i}}$, longitudinal $y^{j_{i}}$, and transverse axis $z^{j_{i}}$ (Figure 2) with three corresponding rotations, namely yaw $\theta^{j_{i}}$, roll $\phi^{j_{i}}$, pitch $\eta^{j_{i}}$. The three axes form the bone local coordinate system originating at the joint $O^{j_{i}}$. In the scope of this paper, we only consider clockwise and counterclockwise rotations of the bones about their transverse axes, i.e., the rotation $\eta^{j_{i}}$ around the $z^{j_{i}}$ axis. As joint *j*_1_ is chosen as a parent joint for articulated transformation, the orientation η^*o*^ about the horizontal axis *x*^*o*^ is equivalent to the joint rotation $\eta^{j_{1}}$, therefore, η^*o*^ is suppressed to avoid redundancy in the pose reconstruction. In total, 5+*N* parameters $\tau =\left\{x^{o},y^{o},z^{o},\theta^{o},\phi^{o},\eta^{j_{i}}\right\}$ are reconstructed, with *i* = 1…*N* and *N* is the number of joints under consideration.

### 2.2. Landmark-based 2D/3D registration

The goal is to align 2D detected landmarks from acquired fluoroscopy images with projections of their 3D reference landmarks to estimate **τ**. In other words, the registration parameters are the result of minimizing the total distances between 2D detected landmarks (*u*^*m*^, *v*^*m*^), and the computed projections of 3D reference landmarks (*u*^*r*^, *v*^*r*^) using **τ** across all *N* joints and *K* landmarks. The estimated parameters $\hat{\tau }$ are defined in (Equation 1):


(1) $$\begin{array}{l} \hat{\tau }=\arg\underset{\tau }{\min}\left\{\overset{N}{\underset{i=1}{\sum }}\overset{K}{\underset{k=1}{\sum }}\omega_{ik}\left({\left(u_{ik}^{r}\left(\tau \right)-u_{ik}^{m}\right)}^{2}+{\left(v_{ik}^{r}\left(\tau \right)-v_{ik}^{m}\right)}^{2}\right)\right\} \end{array}$$

where the distances between the measured and reference landmarks are penalized by different weights ω_*ik*_ based on their hypothesis covariances (23), which will be further discussed in Section 2.4.

To avoid local minima during estimation of the parameters, the object's position and orientation with respect to the acquisition coordinate system are estimated before the joint parameters are reconstructed. The detail process is as follows. First, the projection angle ϕ^*o*^ is adjusted to align the object orientation to the acquisition angle. Next, the object coordinate along the vertical axis *y*^*o*^ is estimated prior to the reconstruction of the three offsets {*x*^*o*^, *y*^*o*^, *z*^*o*^}. After that, the two joint articulation angles $\left\{\eta^{j_{1}},\eta^{j_{2}}\right\}$ are estimated. Finally, the object orientations with respect to the world coordinate system {θ^*o*^, ϕ^*o*^} are estimated. This process is iterated until the loss function evaluation or all the parameter updates are <10^−8^.

### 2.3. 3D landmarks

3D reference landmarks should be key points characterizing the shape of the bones and should be easily distinguishable in the 3D reference model as well as in the 2D radiographs of the whole object. Several methods define 3D reference landmarks based on the 3D model of the object. One of the commonly used methods in computer vision finds a bounding box around the object and uses its vertices as the 3D reference landmarks for registration (28, 29). Peng et al. (23) introduced a technique based on Euclidean distance between voxels and the object's center-of-mass (CoM) to define 3D landmarks of an object given its 3D model. The method avoids involving inaccurate bounding box vertices as the 3D landmarks are drawn from the voxels that belong to segmentation of the 3D object. Although the method showed its advantages over the conventional shape description based on bounding box, there is a risk of choosing 3D landmarks that are too close to each other, resulting in overlap in the 2D radiographs. The reason behind this is that a new landmark was defined as the object voxel with the largest distance to the CoM of the already selected landmarks. The CoM therefore starts to overlap with the original object's CoM, and new landmarks may gather close to the existing landmarks. To solve this problem, we introduce a comprehensive scheme based on the shortest voxel coordinate variance to keep the landmarks distant from the CoM and from each other. Two types of landmarks are determined, namely bounding [similar to (23)] and SIFT (Scale-Invariant Feature Transform) landmarks (24). While the bounding landmarks are selected from ordinary bone voxels, the SIFT landmarks are selected from 3D SIFT keypoints of the bone volume. The landmarks should distribute near/over the bone surface to better characterize its shape and avoid overlapping 2D projections. The shortest coordinate variance scheme is applied to draw the bounding and SIFT landmarks from their initial bone voxels and SIFT keypoints sets, respectively. The scheme to select a list of landmarks from their initial set is as follows:

1. Compute the CoM of the bone segment.
2. Compute 3D coordinate variances of the bone voxels. These variances correspond to the eigen values obtained from principal component analysis (PCA) of the bone voxel coordinates. The smallest and the largest eigen values imply the bone minor and major dimensions, respectively. The landmarks should spread closely to the bone surface to better describe its shape. Therefore, the smallest eigen value σ_*min*_ is used to compute a distance threshold in the later step.
3. Choose the first landmark with the largest Euclidean distance to the CoM. Add the landmark to the list.
4. All the other landmarks *l* are added if their distances to the CoM are largest, and their distances to the existing landmarks *m* in the list satisfy *d*_*lm*_≥λσ_*min*_, with a scale threshold λ chosen heuristically depending on the bone shape and size.

### 2.4. Automatic detection of 2D landmarks with BoneNet

To correctly reconstruct the 3D pose parameters of an animal, 2D landmarks must correspond to 3D landmarks of the reference model and be detected with the lowest possible coordinate errors. Peng et al. (23) trained a deep neural network (PVNet) to automatically detect 2D landmarks in an optical image scene. The 2D coordinates of each landmark were encoded by a voting vector field that points toward the landmark position in the 2D image. PVNet was based on the ResNet18 architecture (30), and obtained by first discarding subsequent pooling layers of ResNet18 when the feature maps were 1/8 size of the original input samples. Then, the fully connected layer was replaced by a convolution layer at the network output. Finally, up-sampling (interpolation) combined with skip connections and convolutions were applied to reconstruct the original image sizes for the bone segments and voting vector fields of the landmarks.

**Figure 3:**
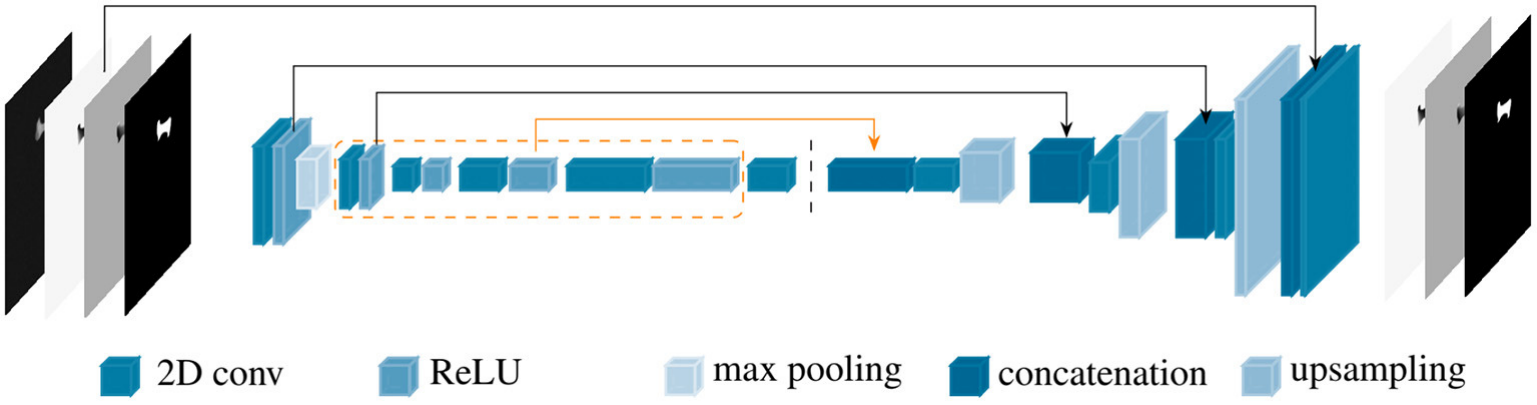
BoneNet, the proposed customized network architecture for automatic 2D landmarks detection of a complex biological object with newly added basic blocks in the convolution blocks (orange dashed lines) and connection (orange arrow). The vertical, dashed line separates the feature learning **(left)** and the interpolation stage **(right)** of the deep network.

PVNet inherited from ResNet18 four main convolution blocks, which were constructed from sequences of basic blocks in the feature encoding stage. Each basic block is formed by two 2D convolutions followed by batch normalization and a ReLU unit. PVNet was designed for accurate inference of only nine landmarks in optical images (23), which resulted in an unstable and slow convergence when applying to X-ray images with a higher number of landmarks. Therefore, the model needs to be adapted to such data. We customized PVNet as follows. New 1, 1, 2, and 1 basic blocks were added to the four convolution blocks in the original PVNet model, respectively. A new connection from the 3*rd* convolution block replaced the shortcut from the 2*nd* convolution block to the first up-sampling layer. The number of features in the subsequent layers were also adjusted accordingly. Figure 3 shows a simplified architecture of the customized network, named BoneNet, with the new basic blocks in the convolution blocks marked by the orange dashed line. The new connection is highlighted with the orange arrow. The rest of the network is identical to the original PVNet architecture. The convolution blocks (left hand side of the dark dashed line in Figure 3) learn and optimize network parameters for image feature extraction. The interpolation layers (right hand side of the dark dashed line in Figure 3) propagate the extracted features and reconstruct original dimensions for the outputs.

**Figure 4:**
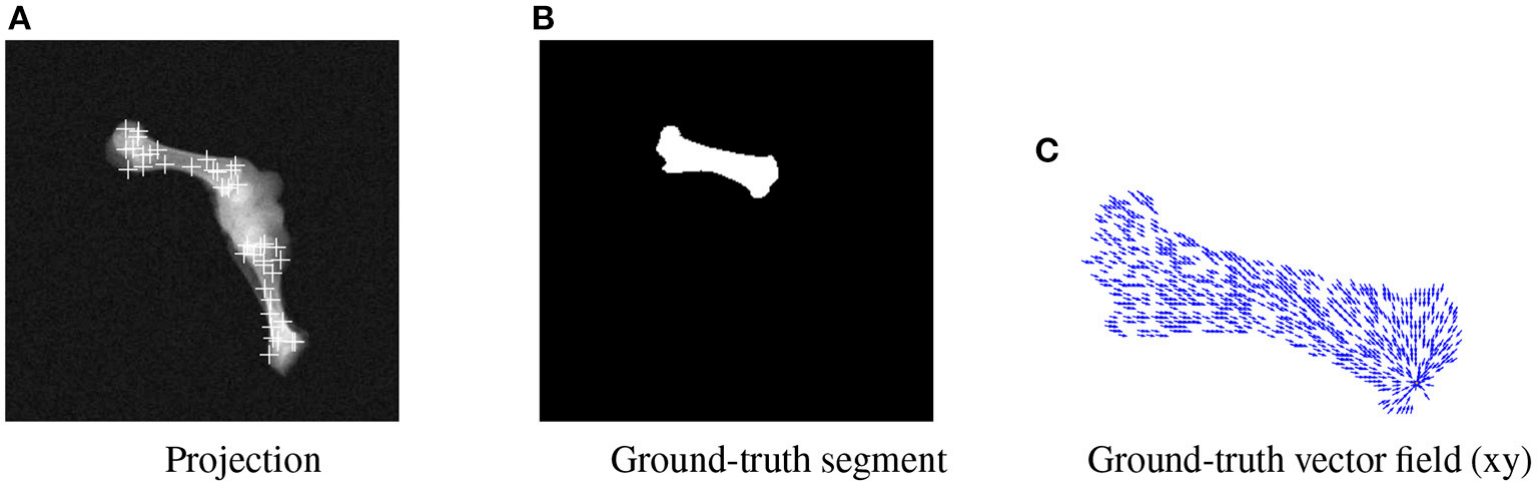
Visualization of an input projection **(A)** with the femur's and tibia's 2D landmarks, a femur segment **(B)**, and the vector field (blue arrows) of a landmark (orange) **(C)**.

BoneNet is trained with a dataset that contains X-ray projections of the bone, corresponding bone binary masks, and 2D ground-truth coordinates of the bone's landmarks. Landmark 2D coordinates are then converted to 2D vector fields as in (23). Figure 4 shows samples of the BoneNet training dataset with 2D landmarks of the femur and the tibia marked by white crosses (Figure 4A), a ground-truth femur segment (Figure 4B), and the corresponding vector field of a landmark (orange star) under the vector form (blue arrow) (Figure 4C). Like PVNet, BoneNet predicts the bone segment and a voting vector field for each landmark of a given input image. The exact coordinates of each landmark are computed from its voting vector field using the voting scheme described in (23). A set of pixel hypotheses is voted for each landmark with corresponding voting scores. Each landmark is then represented by the weighted mean of its hypothesis coordinates $\hat{\mu }$, and a coordinate covariance σ computed as weighted mean squared Euclidean distances between the hypotheses and the mean coordinates $\hat{\mu }$. The Mahalanobis weight ω of the corresponding landmark in Equation (1) is penalized with the inverse of the covariance σ as a higher σ represents a less accurate estimation of the corresponding landmark (23). In general, a training dataset contains input images *I*(*x, y*) with the ground-truth bone segments *M*_*gt*_, and the ground-truth 2D landmark coordinates (*x*_*gt*_, *y*_*gt*_). The learning loss is composed of smooth *L*1 and cross entropy loss ℓ(·) for vector field and segment training, respectively (23). The smooth *L*1 loss is computed as the differences between the 2D predicted *f*[*I*(*x, y*), **ω*_c_***] and the ground-truth vector fields. The cross entropy loss ℓ(·) is computed from the predicted segments *g*[*I*(*x, y*), **ω*_m_***] and the ground-truth segments. BoneNet then optimizes the parameters (**ω*_c_***, **ω*_m_***) to minimize the learning cost $L\left(\omega_{c},\omega_{m}\right)$ (Equation 2).


(2) $$\begin{array}{l} ℒ(\omega_{c},\omega_{m})\\ ={\left\|\left[M_{gt}⊙f\left(I(x,y),\omega_{c}\right)\right]-\left[M_{gt}⊙I(x,y)-(x_{gt},y_{gt})\right]\right\|}_{smoothl1}\\ -\ell \left(M_{gt},g\left(I(x,y),\omega_{m}\right)\right) \end{array}$$

with **ω*_c_, ω_m_*** the learnable weights.

### 2.5. Simulation of articulated transformation

A training dataset comprises X-ray radiographs of the bone, the 2D ground-truth bone segments that contain the landmarks, and the 2D ground-truth coordinates of the landmarks. Training BoneNet requires an extensive, well-labeled dataset, which must be diverse in terms of the landmark relative positions and orientations in the image plane. X-ray images can be simulated from 3D CT volumes of the animal using the ASTRA Toolbox volumetric projector (31, 32).

In principle, one could manually manipulate the joint configuration of the animal sample for every 3D CT scan to generate realistic representations of the animal articulation poses. However, the scanning procedure is time-intensive as a large number of CT scans is needed to cover possible joint configurations. Additionally, the 3D landmarks extracted from each 3D CT scan are inconsistent across the scans due to changes in the object's orientation and position with respect to the scanning volume geometry. A simulation of both rigid and articulated transformations of the animal sample can facilitate this manual procedure. It also maintains the mapping of the 3D landmark coordinates throughout the 3D models as they can be computed with respect to the transformation parameters. In this work, a 3D CT volume of a piglet hindlimb acquired with a high-quality X-ray imaging system, FlexCT (33), is used as the base model for the simulation. The 3D model is with a size of 1, 416 × 1, 416 × 416 voxels, and voxel size of 45μ*m*. It was then downscaled to the size of 850 × 850 × 250 voxels for a more efficient data processing. Then, the rigid transformation of the object with respect to the acquisition geometry is simulated using the ASTRA toolbox vector geometry (31, 32).

Finally, in the articulation transformations, the voxels in the joint areas might undergo more than one affine transformation as the result of consecutive rotations of individual bones relative to the joint local coordinate systems. The resulting transformation is modeled as a weighted fusion of the separate rotations. The weights ω_*f*_(*x*) are obtained as a convolution of a 3D Gaussian kernel with a standard deviation σ_*f*_ and width of *k*_*f*_ sampling rate with the segment volumes of individual bones (Equation 3).


(3) $$\begin{array}{l} \omega_{f}\left(x\right)=g\left(\sigma_{f},k_{f}\right)*V\left(x\right) \end{array}$$

The 3D bone segments are obtained by the following morphological operations in Matlab (34). First, Otsu threshold is applied to remove soft tissues from the original 3D CT model of the limb. Next, small segments with a few voxels are excluded. Only the segments of the bones of interest are retained. Finally, a morphological closing scheme (a dilation followed by an erosion of 25 × 25 × 25 structuring element window) is applied to fill the empty holes inside each segment. The segments are then labeled with a 3D 6-connected component technique.

The Gaussian weights are used in a fuzzy polyaffine fusion scheme introduced by Arsigny et al. (35) to combine individual transformations that occur in a small interval of time 1/*S* with *S* the fusion time scale. Affine transformation of an individual bone includes rotations about its local coordinate system. Principal component analysis (PCA) of non-weighted voxel coordinates is used to define the bone local coordinate system. Three orthogonal eigen vectors $\left(\hat{e_{x}},\hat{e_{y}},\hat{e_{z}}\right)$ represent three bone principal axes, namely the vertical $x^{j_{i}}$, longitudinal $y^{j_{i}}$, and transverse axis $z^{j_{i}}$ (Figure 2). A bone origin is then defined by sliding its CoM along the major semi-axis by the axis length, followed by a visual verification to ensure the origin is at the expected end of the bone. Given a rotation matrix *R*_*t*_, *t* = 1…*M*, with *M* the number of rotations, rotation angle α_*t*_ is computed by:


(4) $$\begin{array}{l} \alpha_{t}=\arccos\left(\frac{tr\left(R_{t}\right)-1}{2}\right) \end{array}$$

with *tr*(*R*_*t*_) the trace of *R*_*t*_. Arsigny et al. (35) defined the transformation speed *A*_*t*_ of rotation *R*_*t*_ as *A*_*t*_ = log(*R*_*t*_), with log(*R*_*t*_) computed by:


(5) $$\log(R_{t})=\left\{ \begin{array}{l} 0\text{ if }\alpha_{\text{t}}\text{= 0 }\\ \frac{\alpha_{t}}{2\sin\alpha_{t}}(R_{t}-R_{t}^{T})\text{ if }\alpha_{\text{t}}\neq \text{0 and }\alpha_{\text{t}}\in (-\pi ,\pi ) \end{array}\right.$$

The 2^nd^-order scheme (35) that computes fusion of *M* individual transformations *R*_*t*_ occur in the time interval 1/*S* is simplified to:


(6) $$T_{2}^{1/S}(x)=x+\frac{\sum_{t}^{M}\omega_{f_{t}}(x)\left(e^{A_{t}/S}-I\right)x}{\sum_{t}^{M}\omega_{f_{t}}(x)}$$

with *x* the object voxel coordinate, *w*_*f*_*t*__(*x*) the fusion weight applied to transformation *t*^th^ of the voxel *x*, and *I* the 3 × 3 identity matrix.

Finally, polyaffine transformation of *x* at *k*^th^ point in time is obtained by taking compositions (°) of *k* sub-transformations $T_{2}^{1/S}\left(x\right)$ (Equation 7).


(7) $$T_{2}^{k/S}(x_{k})=\underset{k\;\text{compositions}}{\underset{︸}{T_{2}^{1/S}(x_{k-1}){}^{\circ}\ldots {}^{\circ}T_{2}^{1/S}(x_{0})}}$$

with 1 ≤ *k* ≤ *S*, and *x*_0_ the initial position of *x*.

Inverse transformation fusion can be obtained by simply taking the opposite of rotation angle α_*t*_. Target voxels are then mapped to source voxels by applying the inverse warping model. As the mapped source voxels are usually non-integer-coordinate-voxels, an interpolation scheme is needed to derive the target voxel intensities afterward. In this work, a 3D cubic-spline interpolation tool is implemented that fits a 3^rd^-order polynomial to the known integer neighboring voxels of an unknown floating voxel to compute its intensity (36). The method is deployed on a GPU infrastructure to increase computational performance as the interpolation is voxel-wise, and a volume usually contains millions of voxels.

## 3. Experiments and results

### 3.1. Training data

**Figure 5:**
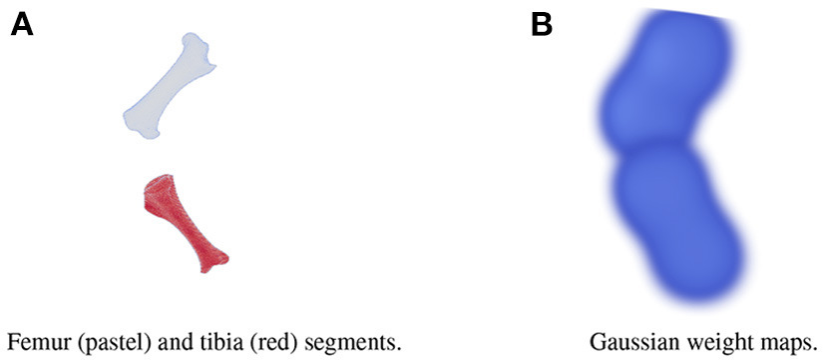
The femur (pastel) and the tibia (red) segments **(A)** of a piglet hindlimb and their Gaussian weight maps **(B)**.

**Figure 6:**
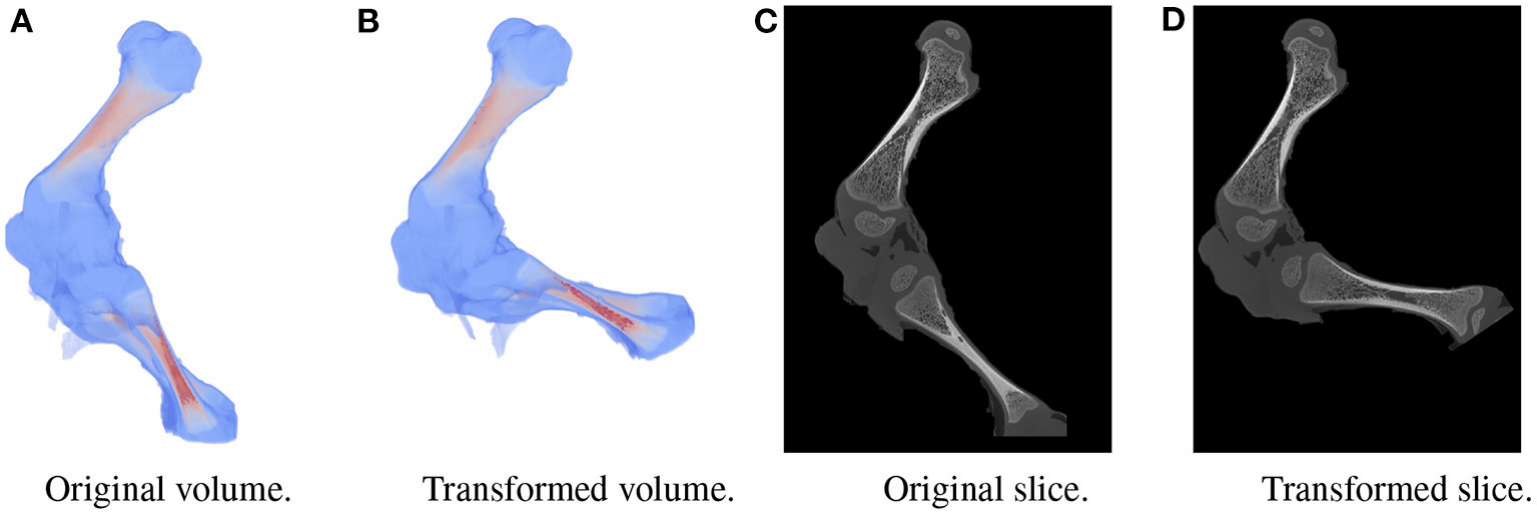
Original volume **(A)** and a the polyaffine transformation fusion **(B)** for rotations of the femur and tibia around their principal transverse axes, with two slices from the original **(C)** and polyaffine **(D)** transformed volume. The two slices do not correspond as the transverse axes are not parallel to the volume coordinate axis.

A large dataset is needed to train BoneNet. The dataset must contain the X-ray images of the hindlimb in different configurations of the bones as well as various limb's positions and orientations with reference to the 2D image space. In the following experiments, all simulation data was generated from a single 3D CT model of a piglet hindlimb sample with muscle removed by dissection. The articulation poses of the limb are simulated using the fuzzy polyaffine fusion scheme discussed in the Section 2.5 with a fusion scale *S* of 18. Fusion weights ω_*f*_(*x*) were computed by the convolution of a Gaussian kernel with σ_*f*_ = 13 and width *k*_*f*_ of 23 with the bone segments. The femur (pastel) and tibia (red) segment of the piglet hindlimb are shown in Figure 5A. Their Gaussian weight maps (Figure 5B) were normalized for fusion of the polyaffine transformations of the individual bones. This allows deformation of the 3D CT model of a piglet hindlimb (Figure 6A) *via* two rotations around the femur and tibia transverse axes (Figure 6B). As shown in Figure 6, the transformed slice structure (Figure 6D) is similar to the source slice Figure 6C. It must be noted that the two slices are not exactly corresponding as the femur and the tibia are rotated around their principal transverse axes, and these axes are not parallel to the volume axis. The smooth transition in the femur-tibia joint area (Figure 6D) demonstrates that our polyaffine fusion and tricubic interpolation can be used for further simulation of the articulated motions of the limb.

**Figure 7:**
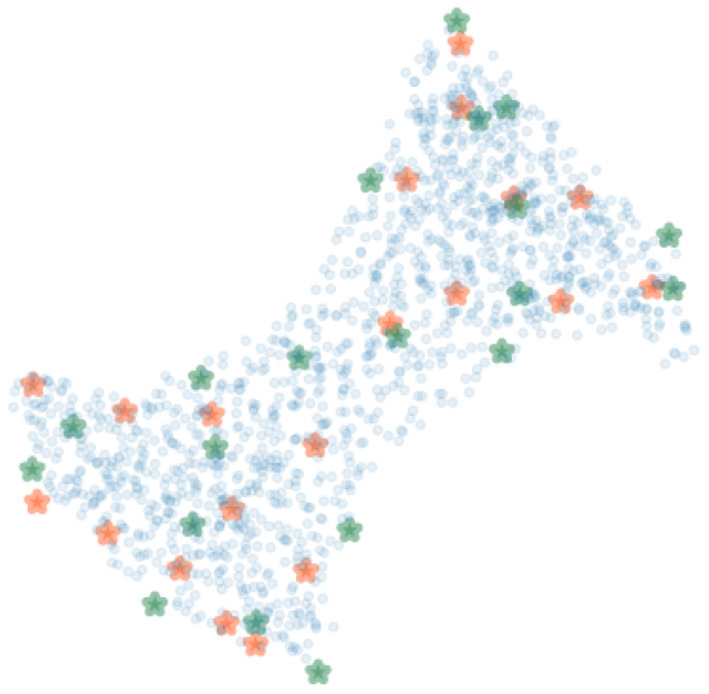
The bounding (green) and SIFT (orange) landmarks of a bone with random bone voxels in blue. The landmarks are distinguishable and at a distinctive distance from each other.

To cover possible poses of the limb, the femur and the tibia were rotated around their transverse axes with six and five equally spaced angles in the range from −30 to 35° and from −20 to 35°, respectively. In total, 30 polyaffine transformed volumes of different articulated poses of the limb were generated. Figure 7 shows 20 bounding (green) and 20 SIFT (orange) landmarks extracted for a piglet femur by applying the shortest coordinate variance scheme presented in Section 2.3. The number of landmarks was chosen heuristically to be 20 for each bone with λ adjusted to (2.1, 3.2) and (2.4, 4.2) for the 3D bounding and SIFT landmark detection of the femur and tibia, respectively. As shown in Figure 7, the landmarks are easily distinguished and distributed close to the surface of the bone. The 3D bounding and SIFT landmarks of the femur and the tibia were also transformed to obtain the corresponding coordinates in the deformed volumes. These volumes were then used for simulation of the 2D X-ray radiographs of the limb with the following scheme.

Rigid positions and orientations of the whole limb with reference to the 2D image space were simulated using the ASTRA Toolbox vector geometry (31, 32). Forty angle intervals were equally sampled from two ranges between −30 and 30° and between 150 and 210°, which replicate the projection angles of a practical acquisition. With each of these angle intervals of ±1.5°, 13 X-ray projections were generated using the ASTRA volumetric projector whose vector geometry is computed with the distances *SOD, SDD* of 6550±180 and 10000±540 voxels, respectively. The limb 3D positions (*x*^*o*^, *y*^*o*^, *z*^*o*^) along the horizontal, vertical and projection axis were modified with ±180, ±120, and ±180 voxel units, respectively. The rigid rotations around two horizontal axes were adjusted to the range of ±15°, and binning factor is set to 6. The 2D projections of the bounding and SIFT landmarks as well as the 2D masks of the corresponding bone segments were also computed using the same geometry and volumetric projector. Additionally, each noiseless projection was scaled to a maximum intensity *I*_0_ randomly generated in a range of 2, 000 ± 350 to diversify the noise level in the simulated dataset. Then, Poisson distributed X-ray projections were simulated by replacing each projection pixel by a random draw from a Poisson distribution with a mean corresponding to the noiseless projection pixel value. In total, the generated dataset contains 15, 600 simulated X-ray radiographs of the piglet limb with ground-truth masks of the bones and the 2D ground-truth coordinates of the 20 bounding and 20 SIFT landmarks of the corresponding bones. The dataset was then shuffled and divided into training, validation, and test sets of 11, 700, 3, 120, and 780 samples, respectively. This test set was used to examine the prediction loss after the training completed. Further study on model inferences were conducted on an independently generated study dataset, which will be discussed in Section 3.3.

### 3.2. Train BoneNet

**Figure 8:**
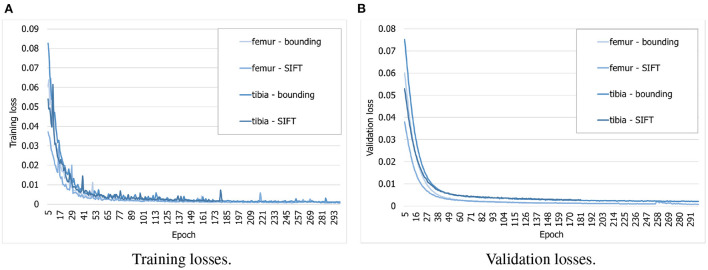
Evolution of training and validation losses over training epochs. All the losses decrease stably and plateau at the final epochs. **(A)** Training losses. **(B)** Validation losses.

To find out whether the customized BoneNet is capable of predicting accurate 2D landmarks in X-ray radiographs, it was trained, validated, and tested on a simulated dataset. BoneNet was trained with a maximum of 600 epochs or until the training and validation losses plateau. Like PVNet (23), the adam optimizer (37) minimizes the smooth L1 loss, which is equivalent to the Huber loss (38), and cross entropy loss [(39), chapter 9] for the vector fields and object segment learning, respectively. A multistep learning rate scheduler (23) that adjusts the base learning rate of 10^−5^ by a multiplication rate of 0.5^*e*^ (*e* the current epoch) was applied for the first five training epochs. Four models were trained individually for 20 bounding and SIFT landmarks of the femur and the tibia. As can be seen in Figure 8A, the training losses descend rapidly over the first 30 epochs and steadily decrease over the rest of the training. The validation losses (Figure 8B) were computed for 3, 120 samples of the corresponding dataset. Although we observe intermittent spikes of the losses throughout the training epochs, overall, both the training and validation losses plateau over the last epochs. The models also do not overfit to the training data as both the losses gradually and stably descend. This is further demonstrated in the numerical evaluation of the 2D landmark detection for a test dataset in Section 3.3. The validation curves (Figure 8B) evolve smoother in comparison to the training losses as the validation points are generally computed after training epoch backpropagations, namely after updates of the model parameters with respect to training batches.

### 3.3. 2D landmarks detection

To study 2D landmark detection accuracy using the BoneNet predicted segments and voting vector fields, we performed a numerical evaluation using a simulated dataset. A study dataset was generated independently from the training set by following procedure. Using the same initial CT volume, we simulated nine articulated volumes with different femur and tibia rotations from the training set. More specifically, three femur ($\eta^{j_{1}}$) and tibia pitches ($\eta^{j_{2}}$) were equally sampled from two ranges between −19 and 30° and between −16 and 30°, respectively. The X-ray images were also generated with a different sampling rate of eight ±7.5°-angle-intervals in the ranges from −30 to 30° and from 150 to 210°. The distances *SOD, SDD* were also manipulated with 6, 450 ± 180 and 10, 200 ± 540 voxels, respectively. The other rigid parameters including {*x*^*o*^, *y*^*o*^, *z*^*o*^}, {θ^*o*^, ϕ^*o*^, η^*o*^} were randomly sampled in the same ranges with the training set.

**Figure 9:**
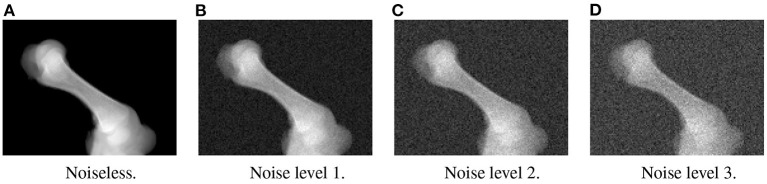
Four sample projections from each of the simulated datasets with noiseless **(A)**, low **(B)**, moderate **(C)**, and high noise level **(D)**.

**Figure 10:**
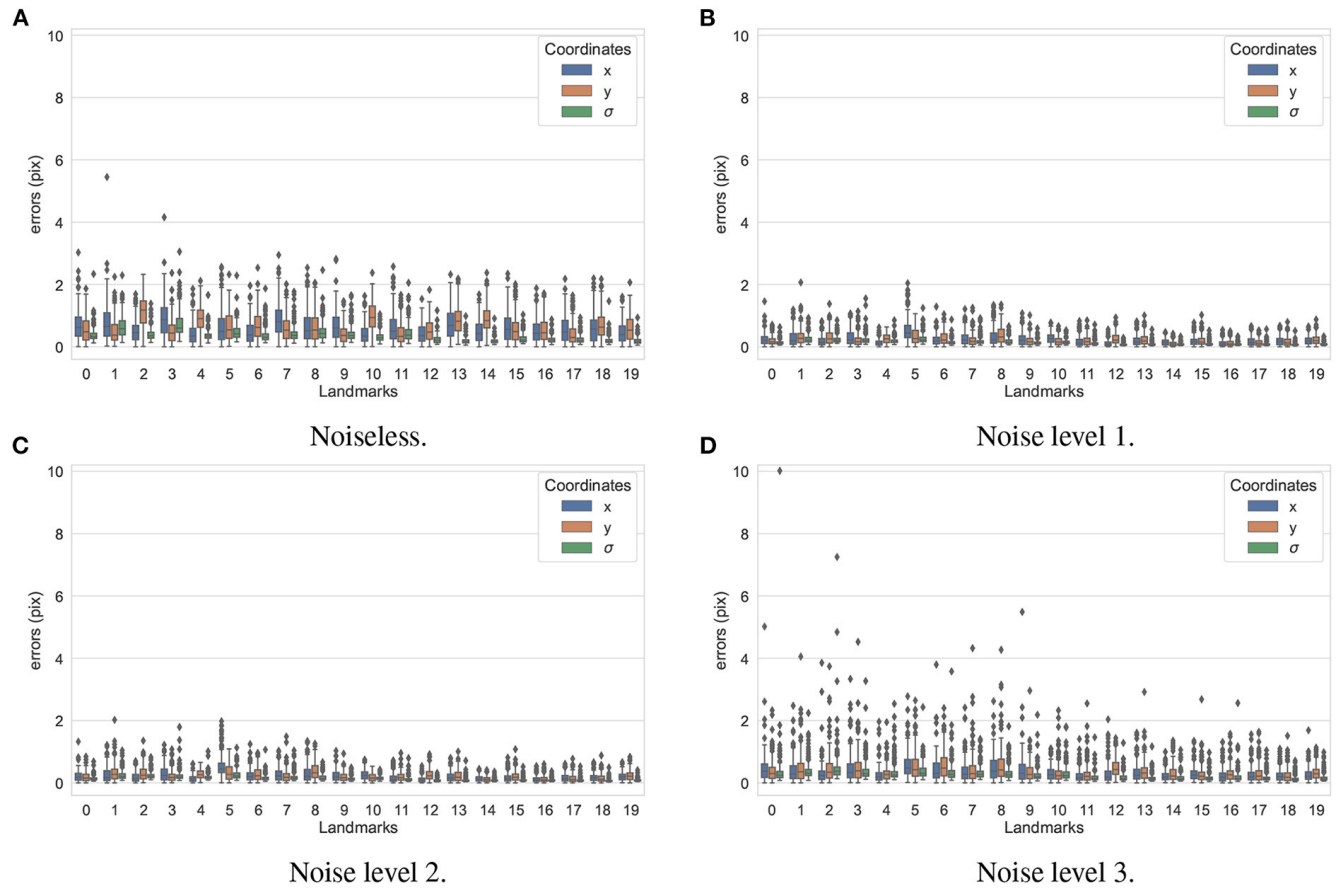
2D landmark extraction errors for four datasets with different noise levels using BoneNet trained with a dataset of noise level 1. With a higher third-quartile of around 1.4 pixels, the noiseless dataset was estimated less accurately than the noisy datasets as they have the third-quartiles of <0.8 pixels. However, there are higher numbers of outliers distributed in broader ranges in the results of noise level 3 (black diamond points in **D**) in comparison to the other three datasets (black diamond points in **A–C**).

In the first experiment, the noise sensitivity of the BoneNet model that was trained with the low noise training dataset was studied. Four datasets of 200 projections were generated with the aforementioned parameters, and the ASTRA toolbox. In addition to a noiseless dataset, three different noise levels were introduced to generate datasets with noise levels 1, 2, 3 corresponding to *I*_0_ of 2, 000±350, 650±150, and 250±50, respectively. Four sample projections are shown in Figure 9 to illustrate the effect of different noise levels on the projection data. Next, 2D landmark detection was performed on the four datasets with the BoneNet model that was trained with the low noise data, (*I*_0_ of 2, 000±350). The method presented by Peng et al. (23) was applied to compute the exact coordinates of each landmark based on its masked voting vector field. Each landmark is represented by a mean 2D coordinate hypothesis $\hat{\mu }$ and a covariance σ. The coordinate errors were calculated as the absolute differences between the ground-truth values, and the inferred mean hypotheses $\hat{\mu }$ for each landmark. The landmark detection errors are summarized in Figure 10. Since the BoneNet model was trained with a dataset of noise level 1, following discussion will use the results obtained for noisy dataset 1 (Figure 10B) as a base line to assess the 2D landmark detection errors. The 2D landmarks in the noiseless dataset were estimated less accurately in comparison to the three noisy datasets as 75% of the samples are estimated with the errors up to 1.4 pixels (upper bars of the blue/orange boxes in Figure 10A). More specifically, for the noise levels 1 and 2, third-quartile error levels of around 0.6 pixels were obtained, while these approximate 0.8 pixels for the level 3 dataset. The higher errors for the noiseless dataset are likely caused by the absence of noiseless samples in the training data. That is, during a training epoch, the forward evaluation of the network learning function (Equation 2) was computed using the noisy data. The learnable network parameters were then updated through the back-propagation process to derive the output feature vectors that best describe landmark positions in a noisy scene. When the trained network was used to infer a landmark in a noiseless image, the output feature map was computed using the same learned parameters. Therefore, it is possible that the feature vector is not mapped correctly to the expected position of the landmark. The relatively low inference accuracy of a deep neural network (trained with noisy data) on a noiseless or less noisy testing dataset has also been reported in other studies (40–42). More experiments are needed to analyze BoneNet's performance on noiseless data and data with different noise levels in both training and testing dataset. This experiment also demonstrates that, although having a relatively higher noise level (Figure 9D) compared to the level applied to the training dataset, the BoneNet model is still capable of detecting 2D landmarks at noise level 3, albeit with a slightly reduced accuracy. We also observe outliers with larger coordinate inaccuracies for noise level 3 as the error levels are up to 10 pixels, and more number of landmarks detected with errors of around and above 4 pixels (black diamond points in Figure 10D) in comparison to the results for noise levels 2 and 3 (black diamond points in Figures 10B,C). However, the results generally indicate that, if BoneNet is trained with a similar noise level to the testing or real data, the model would be robust to noise, and could tolerate a broad range of noise levels. Furthermore, adding noise to the training data is also considered as a data augmentation technique that could reduce overfitting, and help the model cope with noise in the real data [(43), chapter 7] (44, 45).

**Figure 11:**
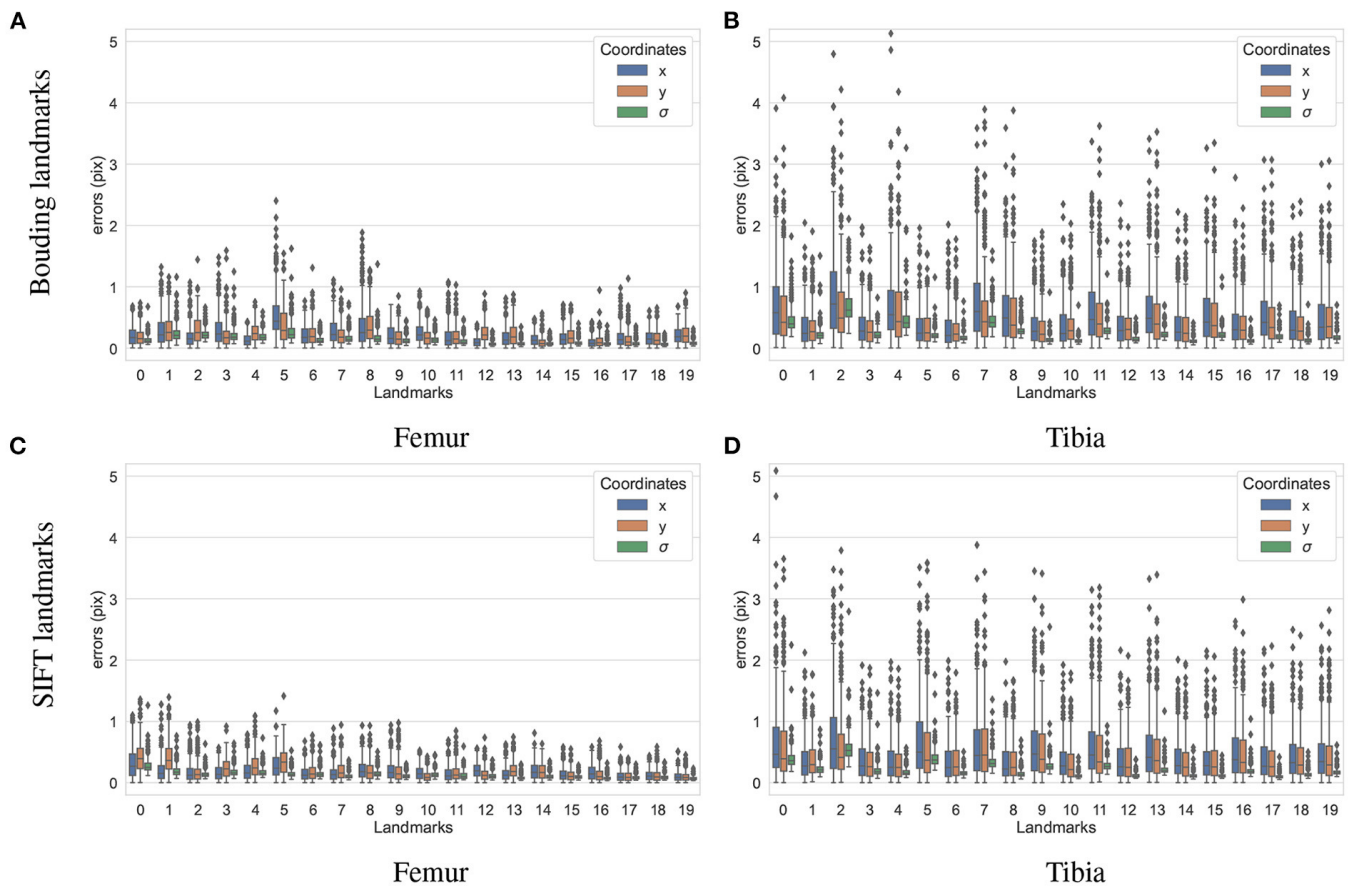
Visualization of the estimated 2D landmark coordinate errors. The femur landmarks **(A,C)** are detected with lower coordinate errors as the third quartiles and maxima are around 0.5 and 1.2 pixels and both lower than 1.1 and 2.2 pixels of the tibia landmarks **(B,D)**.

To test how accurately the landmarks were detected for the different bones and landmark types, another dataset containing 200 X-ray projections was simulated using ASTRA toolbox volumetric projector. The four trained BoneNet models infer the landmark voting vector fields and the bone binary masks in the study X-ray radiographs for two type of bones (femur and tibia) and landmarks (SIFT and bounding). The femur's landmarks are estimated more accurately than the tibia's as the respective upper whiskers (vertical, black lines of blue/orange boxes) extend to 1.1 and 2.5 pixels, and inter-quartile ranges (blue/orange box areas) are around 0.1–0.6 and 0.2–1.1 pixels (Figure 11). Median coordinate errors are up to 0.3 and 0.6 pixels for the femur and tibia landmarks, respectively, demonstrating that 50% of the landmark samples are estimated lower than these errors. Although all landmarks are detected with a median error of less than 0.6 pixels, several landmarks tend to be less accurately estimated than the rest, such as the fifth of the femur bone (Figure 11B). As the covariance measures (σ) are proportional to the error levels of the corresponding landmarks (blue/orange), the higher the covariance, the less confident the estimated landmark coordinates. Consequently, the less accurately detected landmarks are weighted less in the pose reconstruction cost function (Equation 1).

### 3.4. 3D pose reconstruction

**Figure 12:**
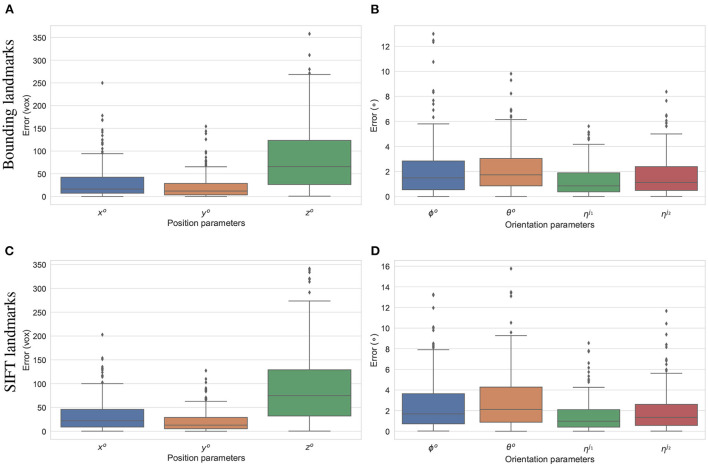
Estimation errors of the pose parameters using the bounding **(A,B)** and SIFT **(C,D)** landmarks. The estimations using the bounding return lower error ranges in comparison to the results using SIFT landmarks, especially for the rotation parameters **(B,D)**.

The final experiment is to study how the predicted 2D landmarks perform in 3D pose reconstruction for the study samples. The voted landmarks were used to estimate the 3D pose parameters with two joint rotations (the femur and the tibia) $\tau =\left\{x^{o},y^{o},z^{o},\theta^{o},\phi^{o},\eta^{j_{1}},\eta^{j_{2}}\right\}$. *SOD*, *SDD*, and η^*o*^ are fixed to the ground-truth values, and all the other parameters are initialized to 0. A numerical study was performed for the reconstruction of 3D poses of the 200 simulated samples. The results are summarized in Figure 12. The offsets of the limb with reference to the horizontal axis parallel to the detector plane (*x*^*o*^), and the vertical axis (*y*^*o*^) are estimated with median errors of around 20 voxel units indicating that errors of 50% of the samples lower than this value. Since, the magnification factors were simulated around 1.5, the projection of a point can be 30 voxels units offset from the correct position. However, the binning factor is 6, so the offset approximates to five pixels. The limb position along the projection axis *z*^*o*^ is estimated with a median of 60 voxels, and 75% of the samples having *z*^*o*^ error of less than 125 pixels (middle and upper bars of the green boxes in Figures 12A,C, respectively). If the respective simulated distances of *SOD, SDD*, which are 6, 450±180 and 10, 200±540 voxels, are accounted for, the computed error makes up around 2% of the projection magnification. Therefore, this gap is hardly visible in the projected image in terms of pixel positions. As shown in Figures 12B,D, 50% of the samples are with the rigid {θ^*o*^, ϕ^*o*^} and the articulated $\left\{\eta^{j_{1}},\eta^{j_{2}}\right\}$ rotation errors below 1.9 and 0.9°, respectively. The rigid rotations {θ^*o*^, ϕ^*o*^} of 75% of the test samples are reconstructed more accurately using the bounding landmarks, with an error of 3° in comparison to 4° for the SIFT landmarks (upper bar of the blue/orange boxes in Figures 12C,D. The articulated rotations $\left\{\eta^{j_{1}},\eta^{j_{2}}\right\}$, have lower third quartile levels of around 2° using either the bounding or SIFT landmarks as demonstrated in Figures 12C,D, upper bars of the green/red boxes. In general, the rotation parameters reconstructed with the bounding landmarks are more accurate as the upper whiskers and interquartile ranges are lower than the results using the SIFT landmarks (Figures 12C,D).

**Figure 13:**
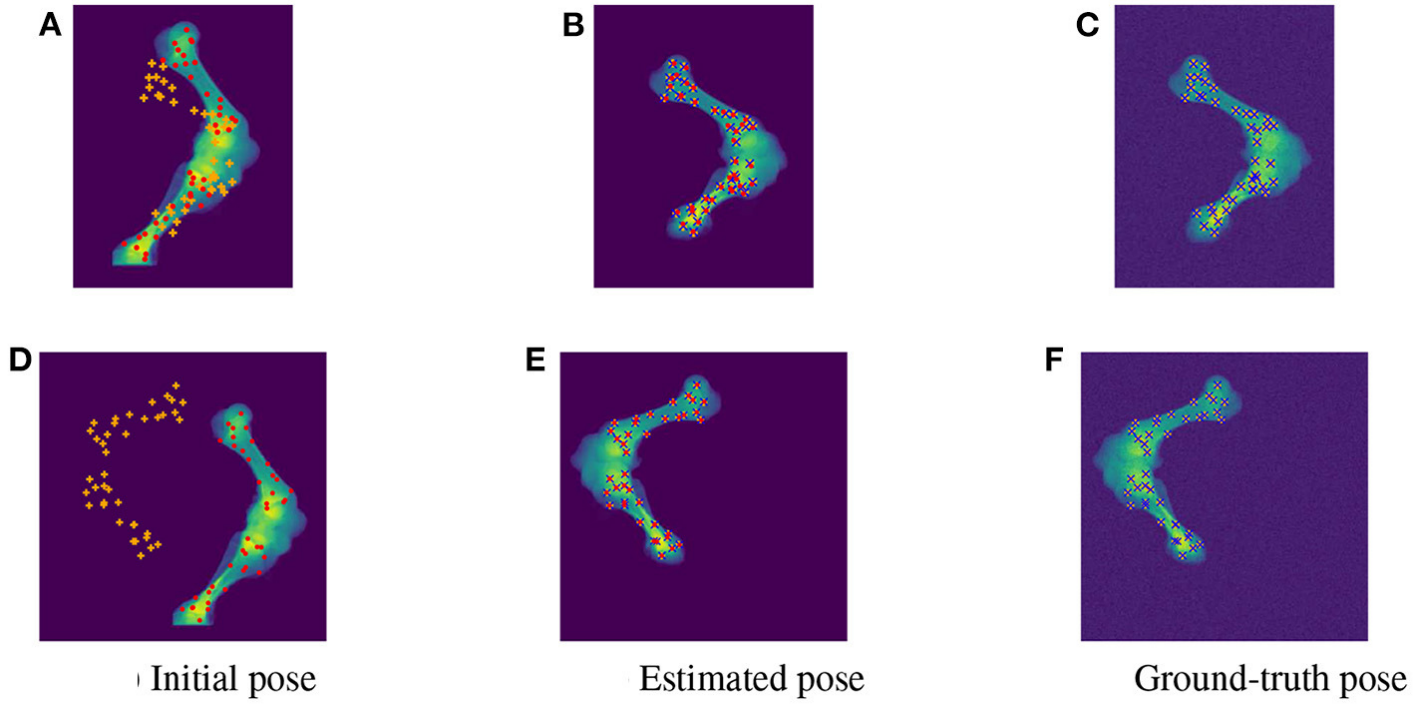
Visualization of the 2D views for the initial **(A,D)**, estimated **(B,E)**, and ground-truth **(C,F)** poses of the two test samples with predicted (orange), ground-truth (blue), and reconstructed (red) landmarks. The 2D detected landmarks (orange) are not aligned with the computed landmarks (red) in the initial poses **(A,D)**. After registration, the estimated and detected landmarks align with the bones, however, the reconstructed landmarks (red) of the high error sample do not always overlap the detected landmarks (orange) **(B)**. This is not the case with the accurate registered sample as the ground-truth (blue), detected (orange), and registered (red) landmarks are aligned correctly **(E)**.

**Figure 14:**
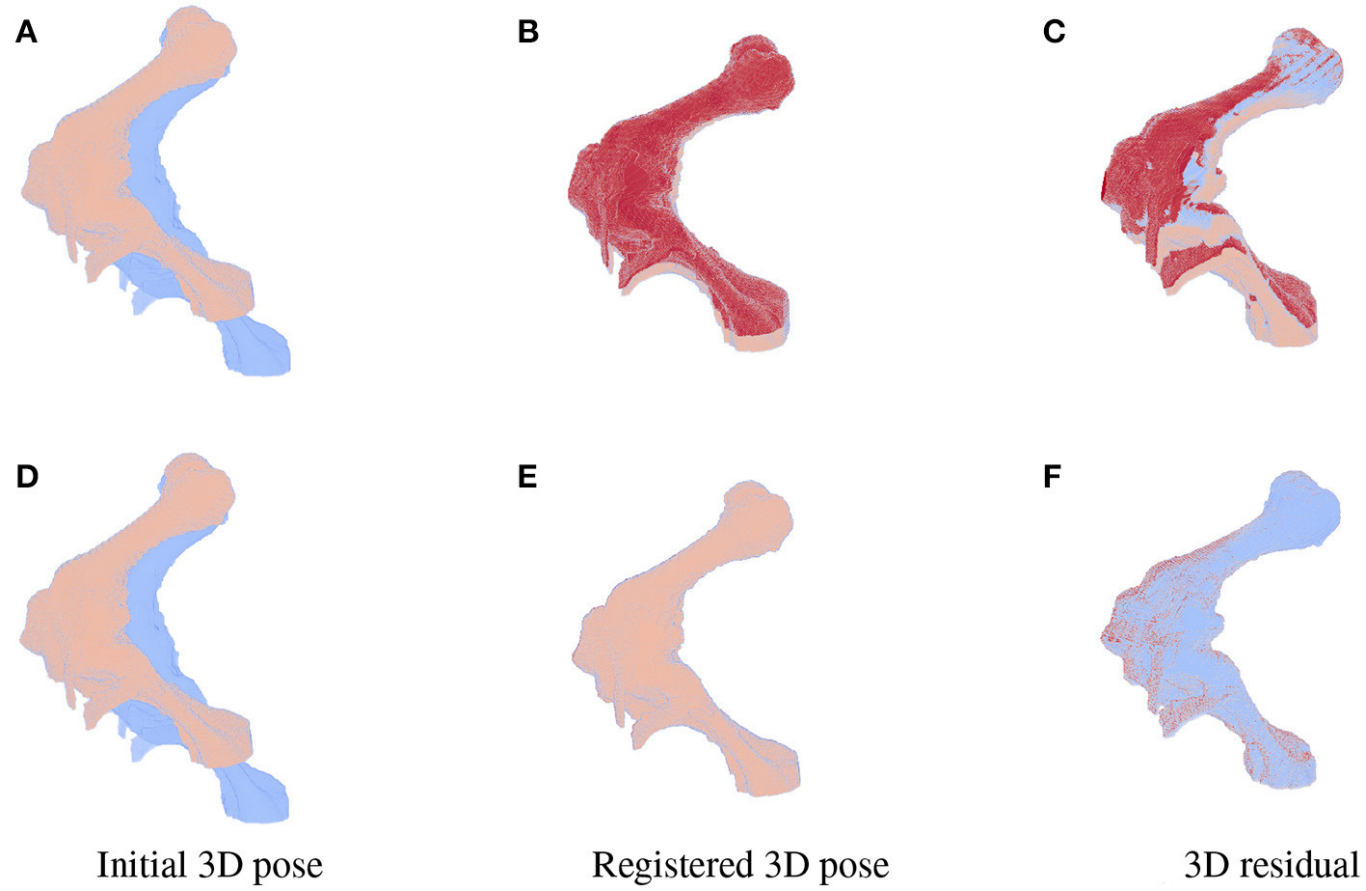
3D views for the registration of the 3D pose of the limb (orange) with reference to the original volume (blue) for the high (first row) and low (second row) pose reconstruction errors. Initial reference (orange) and original (blue) poses are shown in **(A,D)**. With the high error, the 3D estimated volume (red) does not completely overlap the target volume (orange) **(B)** and the gaps are clearly visible as the red and orange volume residual in **(C)**. Accurate estimate of the parameters can be observed in **(E,F)** with overlapping on most of the ground-truth and registered volume [blue region in **(F)**], only minor gaps are seen as the remaining darker spots scattered over the residual volume.

**Figure 15:**
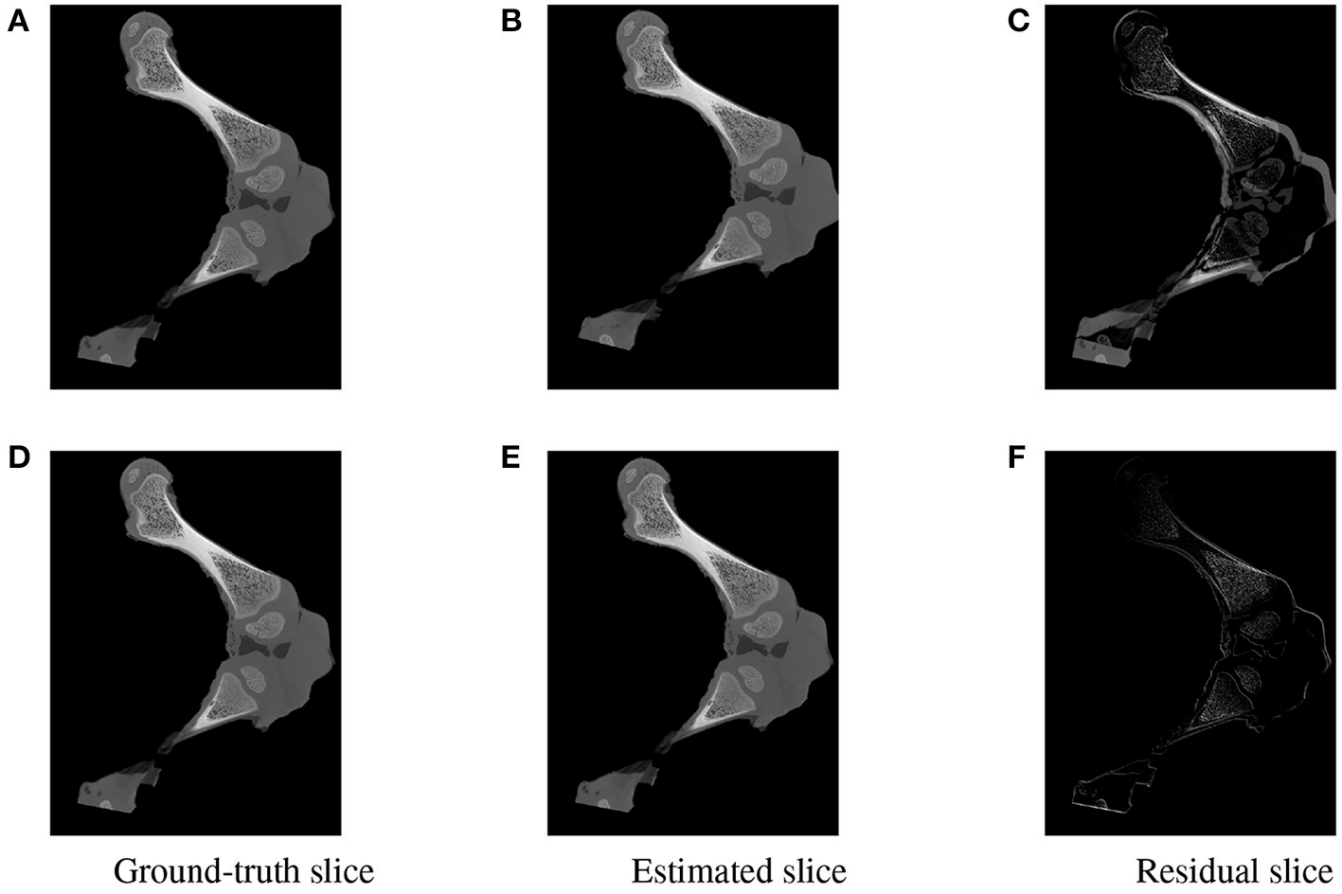
Visualization of the slices extracted from the same positions in the 3D ground-truth **(A,D)** estimated **(B,E)** volumes of the two testing samples. The difference between the corresponding ground-truth and estimated slices are shown as residual images in **(C,F)**. The residual of the high error sample **(C)** is more apparent with a strong magnitude in comparison to a minor and less visible gap for the accurate registered sample **(F)**.

As the parameters were estimated with notable numerical errors, we conducted a further visual inspection for two typical test samples whose errors situated in the upper (high error), and lower (low error) whisker areas of Figure 12. The results associated to the high and low error sample are shown in the first and second row of Figures 13–15, respectively. First, 2D views of initial (Figures 13A,C), reconstructed (Figures 13B,D), and ground-truth (Figures 13C,F) poses for the two test samples are visualized in Figure 13. The ground-truth, detected, and registered landmarks are highlighted in blue, orange, and red, respectively. As can be seen in the first column of Figure 13, both the initial orientation and position of the limb do not match the detected landmarks (orange). After the registration (Figures 13B,E), the detected landmarks (orange) are aligned with the bone and close to the reconstructed landmarks (red). While the landmarks computed with high-error parameters do not always overlap the detected and ground-truth landmarks (Figure 13B), the low-error computed landmarks are well-aligned with the detected ones (Figure 13E). The inaccurate parameters also pose a visible gap in the tibia projection between Figures 13B,C. With well reconstructed parameters, no difference can be seen in the estimated and ground-truth projections shown in Figures 13E,F. Then, registration errors are shown in 3D to give an insight into the estimation of the femur and tibia rotation around their transverse axes $\left\{\eta^{j_{1}},\eta^{j_{2}}\right\}$. The corresponding 3D views for the two testing samples are shown in Figure 14 with the reference, ground-truth (target), and registered pose are in blue, orange, and red, respectively. Before 3D registration, the orientation of the limb (blue) is misaligned with the target pose (orange) (Figure 14D). When the estimated articulation angles $\left\{\eta^{j_{1}},\eta^{j_{2}}\right\}$ are applied to transform the original reference volume, the registered volumes (red) overlaps with the target volumes (orange) (Figures 14A,E). However, as having inaccurate estimated parameters, there is a visible gap between the ground-truth and registered volume at the lower end of the tibia bone (orange) in Figure 14B. Volume registration distances were computed and shown in Figures 14C,F with the orange, blue, and red regions representing the residuals of the ground-truth, overlap, and estimated volume, respectively. The registration error is clearly visible as the ground-truth (orange) and reconstructed (red) residual in Figure 14C, while with an accurate estimate of the parameters, there is only a marginal gap between the reconstructed and the ground-truth 3D poses marked by non-blue regions in Figure 14F.

To further inspect the visual impact of the registration errors, two slices were extracted at the same position from the ground-truth and reconstructed volumes for each of the two test samples. Registration residuals were computed between these slices and the results are summarized in Figure 15. The gap is clearly visible with high magnitude of misalignment in the residual slice of the high error sample Figure 15C. However, a marginal residual is observed in the accurate pose reconstructed sample Figure 15F.

## 4. Discussion

In this paper, we introduced a comprehensive landmark-based method for 2D/3D registration to reconstruct the 3D pose of an object using its fluoroscopic X-ray image and a 3D reference model. The method aligns the 2D detected landmark positions in the X-ray image with the 2D projections of corresponding 3D landmarks. As previous 3D landmark selection methods are prone to overlapping projected landmarks, a shortest coordinate variance scheme was developed to detect the potential 3D reference landmarks. With the shortest coordinate variance threshold, the 3D landmarks were distributed over the object surface and at a distinctive distance from each other. This scheme facilitated distinguishing the 3D landmarks in the reference models as well as detecting the 2D landmarks in the 2D fluoroscopy images.

The conventional landmark extraction methods do not allow to easily map the 2D detected landmarks to the 3D reference landmarks for an accurate alignment of the object. Therefore, we introduced a deep learning method to overcome this obstacle. In general, a trained deep learning model with a well-labeled dataset can predict the positions of the 2D landmarks in a 2D X-ray radiograph. Although there are various deep learning models introduced for landmark detection and registration, a deep neural network that fits our specific object (piglet limb) and the number of landmarks to be detected was not available off the shelf. One of the most relevant models is PVNet (23), which was introduced to detect 2D landmarks in optical images. PVNet originally tackled occlusion in visible light photography. This model was designed to handle only nine 2D landmarks in the scenes. However, our preliminary experiments for the limb data suggested having less than 20 landmarks is insufficient to reconstruct the 3D poses of the limb using a single X-ray radiograph. Therefore, we herein presented BoneNet, which is inspired by PVNet, to adapt to a higher number of landmarks and a more complex biological object. By adding five more convolution basic blocks to the feature encoding stage in the original PVNet, BoneNet was capable of robustly extracting feature vectors from the X-ray imaging data and propagating the features toward upscaling layers. A shortcut from a feature encoding layer to an interpolation layer was replaced to transfer more feature vectors to the output and derive more landmarks. The simulation results show that BoneNet was able to accurately detect the 2D landmarks in simulated, noisy 2D X-ray images. The numerical evaluation for pose reconstruction using the detected landmarks demonstrated promising rigid and articulated parameter estimations. However, further study is needed to clarify the source of errors as well as to minimize the residual errors in both 2D landmark detection and 3D pose reconstruction.

The neural network training requires a large amount of diverse labeled data in terms of the object's positions, orientations, and articulation poses. Therefore, we also applied the polyaffine fusion scheme (35) for a realistic data simulation. An inverse transformation and a 3D tricubic spline interpolation module were also implemented for a smooth and continuous 3D volume transformation. This module and the ASTRA Toolbox (31, 32) served as a data curation tool to prepare the BoneNet training dataset, and the validation and test data to evaluate our whole registration method as the ground-truths were known. We were also able to compute the 3D landmark positions consistently across the transformed 3D volumes by using the same transformation model and parameters.

In the scope of this paper, we considered only a single piglet limb from which the muscle was dissected as a test object. Such simulation neglected the presence of muscle and other types of soft tissue in a real animal model that would certainly complicate the 2D landmark detection. Therefore, in future work, an evaluation of our proposed method with more complex objects, including limbs with muscles, soft tissues, and ultimately, a whole piglet model, is needed. A whole limb study would include acquiring CT scans of the limb to use as a reference model, followed by 3D landmark extraction, simulation of the 2D X-ray datasets, as well as training, and evaluation with the new data. Moreover, the current noise simulation considers neither X-ray source model nor detector responses. Although the preliminary results indicate a high robustness to noise, a further study is necessary to train and to evaluate the performance of BoneNet at the noise level of a real X-ray fluoroscopy system (46). As an alternative, specialized denoising methods could be applied directly to the acquired fluoroscopy images prior to the 2D landmark inference. Such studies are the prerequisite steps toward the evaluation of BoneNet on 2D landmark detection in real X-ray fluoroscopy radiographs. Furthermore, in our current implementation, a deep neural model was trained specifically for each landmark type (bounding, SIFT) and bone (femur, tibia). This training technique is inefficient as a more complex object requires numerous models to be trained. Therefore, we plan to improve the current BoneNet architecture to learn and predict different types of landmarks and bones using a single training model. Our current technique uses only a single cone-beam X-ray radiograph for 3D pose reconstruction. In the future, we intend to employ X-ray images from a biplanar X-ray scanner [e.g., (47)], to gain the accuracy of the 3D pose parameter estimation as more geometric information is taken into account. We also aim at evaluating the method with real X-ray fluoroscopy images for a complete reconstruction of the piglet 3D locomotion.

## 5. Conclusion

In general, our proposed method tackled the difficulties in generating a well-labeled training dataset for 2D landmark detection using a manual approach. Our method employed an automated procedure to robustly detect 3D landmarks compared to the CoM-based technique (23). The computed 3D landmark coordinates across the transformed volumes allowed computing the 2D landmark positions accurately for the training dataset. This procedure also eliminates human errors in manual landmark annotations. The customized PVNet architecture (BoneNet) showed stable convergences over the training with two types of landmarks and a biological sample. The inferences of the bone segments and landmark vector fields with BoneNet resulted in accurate detection of the 2D landmarks in X-ray data from which the 3D poses of the object could be accurately reconstructed.

## Data availability statement

The raw data supporting the conclusions of this article will be made available by the authors, without undue reservation.

## Ethics statement

The animal study was reviewed and approved by the Ethical Committee for Animal Testing of the University of Antwerp, Belgium (ECD 2015-26).

## Author contributions

VN contributed to design and implementation of the method, draft writing, and editing the manuscript. LA, ZL, FM, and JV contributed to the design of the method. JS and JDB contributed to supervision and conceptualization of the study. All authors contributed to manuscript writing, revision, and approved the submitted version.

## Funding

This work has been supported by the University Research Fund, UAntwerp BOF-GOA 2016 33927, the Research Foundation—Flanders (FWO) SBO project MetroFlex (S004217N), the FWO SBO project (S003421N), the European Commission through the INTERREG Vlaanderen Nederland program project Smart^*^Light (0386), and the Flemish Government under the Onderzoeksprogramma Artificiele Intelligentie (AI) Vlaanderen program.

## Conflict of interest

The authors declare that the research was conducted in the absence of any commercial or financial relationships that could be construed as a potential conflict of interest.

## Publisher's note

All claims expressed in this article are solely those of the authors and do not necessarily represent those of their affiliated organizations, or those of the publisher, the editors and the reviewers. Any product that may be evaluated in this article, or claim that may be made by its manufacturer, is not guaranteed or endorsed by the publisher.
